# Supplementary material for: Slow oxidation of magnetite nanoparticles elucidates the limits of the Verwey transition
Source: Nat Commun. 2021 Nov 4;12:6356. doi: 10.1038/s41467-021-26566-4 (PMC8568917; doi:10.1038/s41467-021-26566-4)
Supplement: Supplementary file 1 — Supplementary Information [file 41467_2021_26566_MOESM1_ESM.pdf]

Supplementary Information

Slow oxidation of magnetite nanoparticles elucidates the limits of the Verwey transition

Taehun Kim[1,2], Sangwoo Sim[2], Sumin Lim[3], Midori Amano Patino[4], Jaeyoung Hong[5,6], Jisoo Lee[5,6], Taeghwan Hyeon[5,6], Yuichi Shimakawa[4], Soonchil Lee[3], J. Paul Attfield[7], and Je-Geun Park[1,2,8,*]

[1]Center for Quantum Materials, Seoul National University, Seoul 08826, Republic of Korea

[2]Department of Physics & Astronomy, Seoul National University, Seoul 08826, Republic of Korea

[3]Department of Physics, Korea Advanced Institute of Science and Technology, Daejeon 34141, Republic of Korea

[4]Institute for Chemical Research, Kyoto University, Kyoto 611-0011, Japan

[5]Center for Nanoparticle Research, Institute for Basic Science, Seoul 08826, Republic of Korea

[6]School of Chemical and Biological Engineering, Seoul National University, Seoul 08826, Republic of Korea

[7]Center for Science at Extreme Conditions and School of Chemistry, University of Edinburgh, Edinburgh EH9 3JZ, United Kingdom

[8]Institute of Applied Physics, Seoul National University, Seoul 08826, Republic of Korea

* Corresponding author: jgpark10@snu.ac.kr

Supplementary Figures

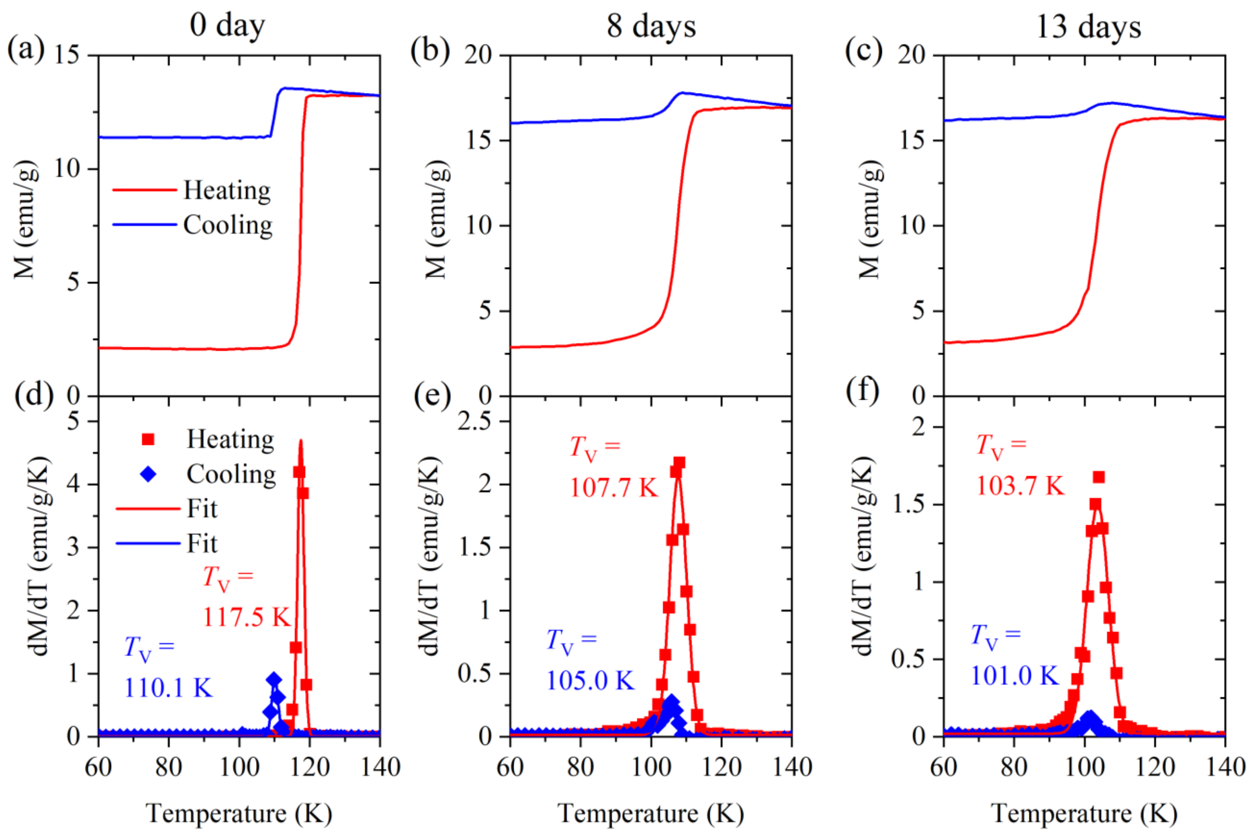

Supplementary Figure 1. Thermal hysteresis of the Verwey transition is suppressed during the oxidation process. (a-c) The magnetization curve taken during heating and cooling are plotted at each oxidation time, 0, 8, and 13 days, respectively. All data were measured at H = 100 Oe. (d-f) The first derivatives of the magnetization (dM/dT) are shown. A Gaussian function was used to fit the peak in dM/dT.

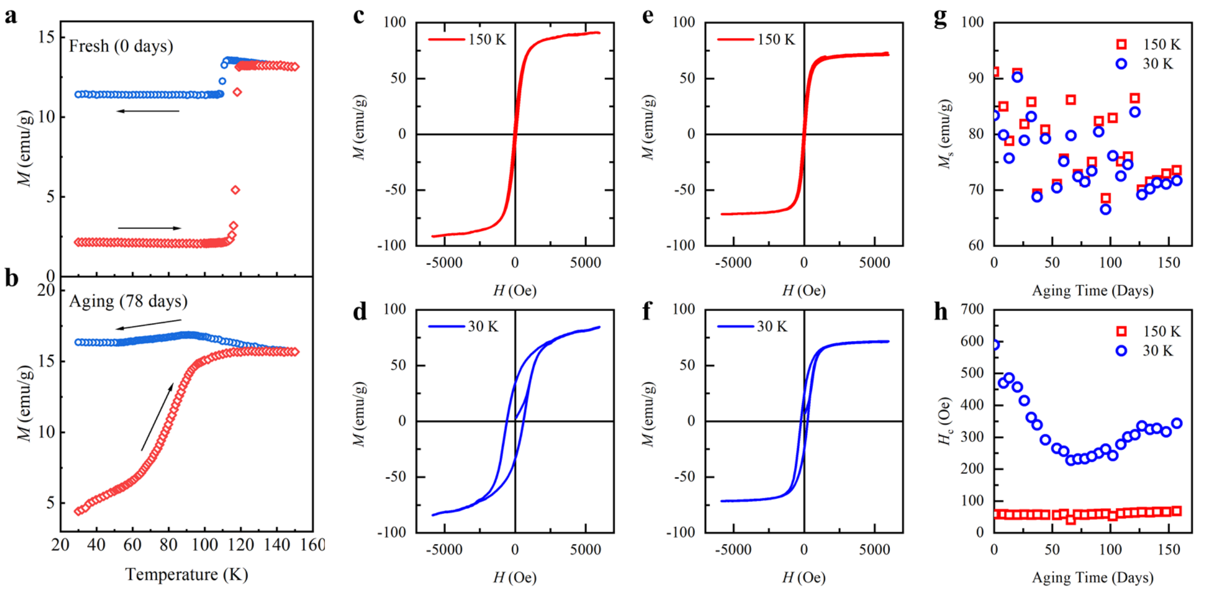

Supplementary Figure 2. The magnetization for the fresh and oxidized (78 days) samples. (a,b) The red(blue) curve indicates the data taken during heating(cooling). The thermal hysteresis of 10 K is observed for the fresh sample, while the thermal hysteresis is absent for the oxidized (78 days) sample. The data were measured at $H = 100$ Oe. (c,d) The isothermal magnetization $M(H)$ curve for the fresh sample. (e,f) The isothermal magnetization $M(H)$ curve for the oxidized (78 days) sample. (g) The saturated magnetization (M_s) as a function of aging time. Both M_s taken at 30 and 150 K gradually decrease upon oxidation. (h) The magnetic coercive field (H_c) as a function of aging time. H_c taken at 30 K shows an initial suppression and a later upturn during the oxidation as identical to the behavior from T_v, whereas H_c taken at 150 K remains constant.

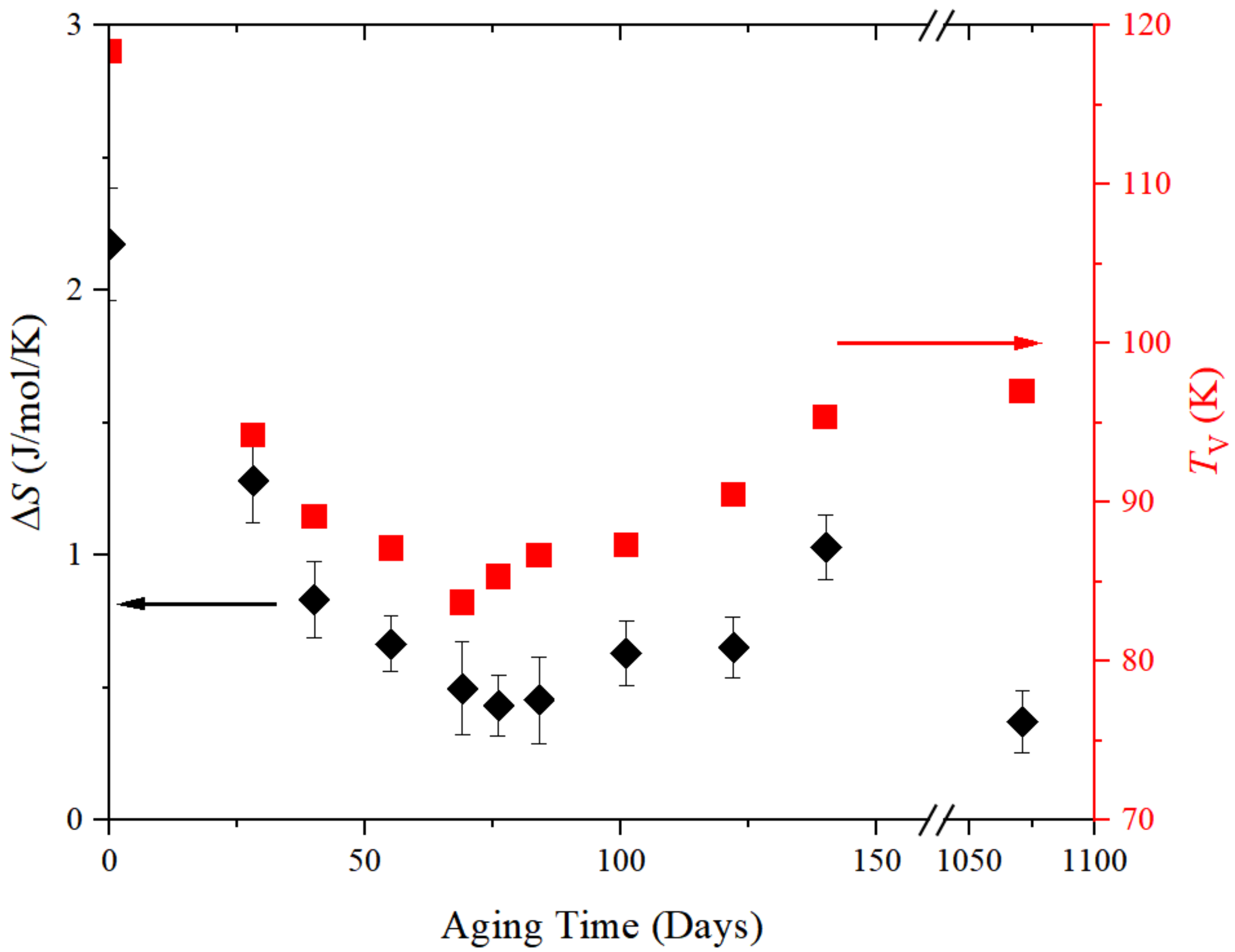

Supplementary Figure 3. The estimated entropy changes through the Verwey transition and T_V as a function of aging time. (Left) The entropy changes were estimated by integrating the magnetic heat capacity data after subtracting the background signals as discussed in Methods. The error bars are defined by the standard deviation from the fitting results of background subtraction. (Right) The extracted T_V from the heat capacity data as a function of oxidation periods.

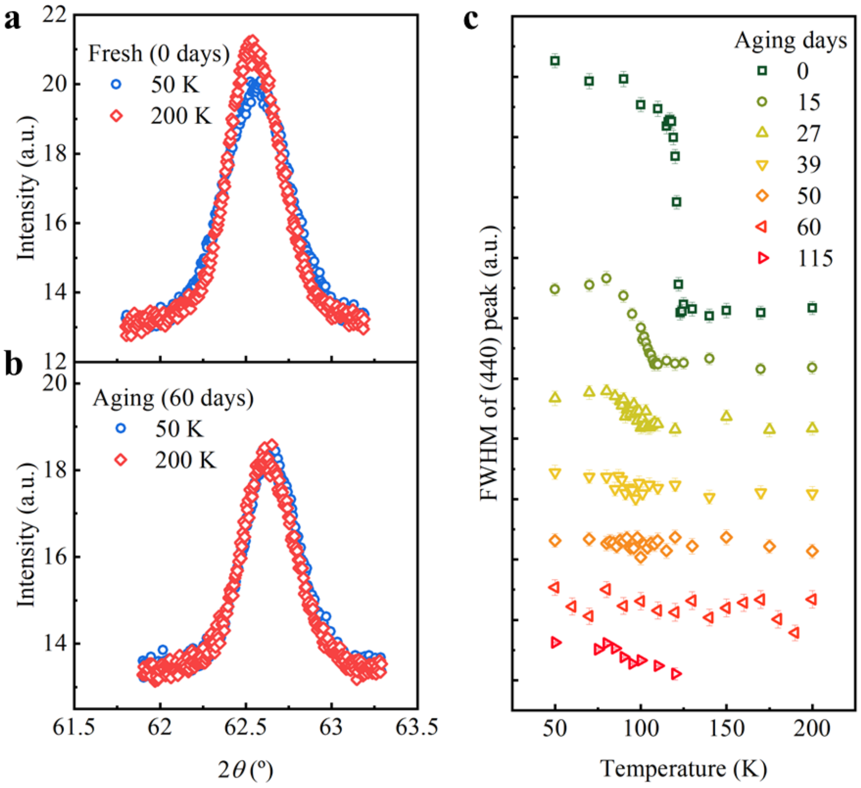

Supplementary Figure 4. The (440) XRD peak was measured at 50 and 200 K for the fresh and oxidized (60 days) sample. (a,b) For the fresh sample, the FHWM of the peak and the height of the peak change through the Verwey transition. However, there is little change for the oxidized case. (c) The stacked plots for the FHWM of the (440) XRD peak. The FWHM is obtained by fitting the XRD peak using a Gaussian function.

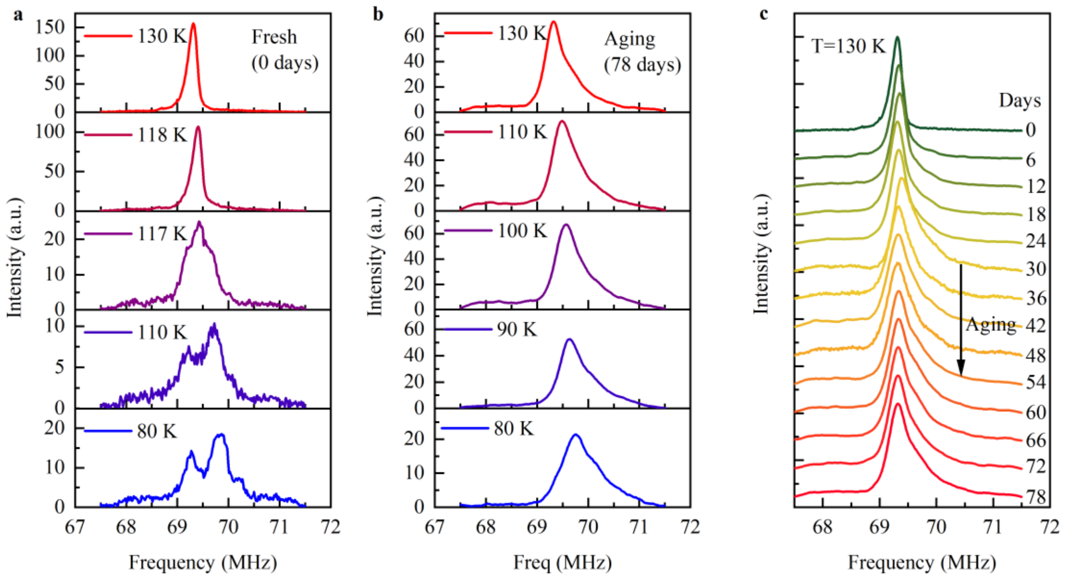

Supplementary Figure 5. The ^{57}Fe NMR spectra were taken at various temperatures. (a,b) Data for the fresh and oxidized (78 days) sample. (c) The stacked plots for the NMR spectra were taken at 130 K.

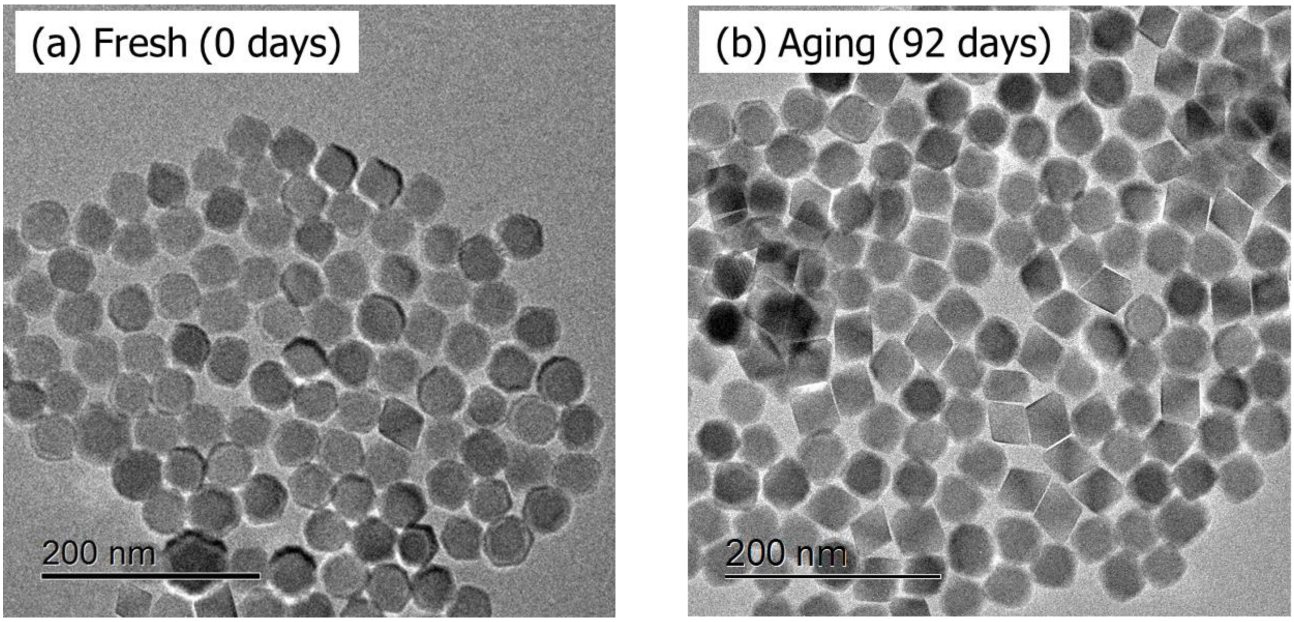

Supplementary Figure 6. The TEM images of fresh and aged magnetite samples. (a) fresh and (b) oxidized (92 days) samples. Within the experimental resolution, there is no visible change in the shape or size of nanoparticles.

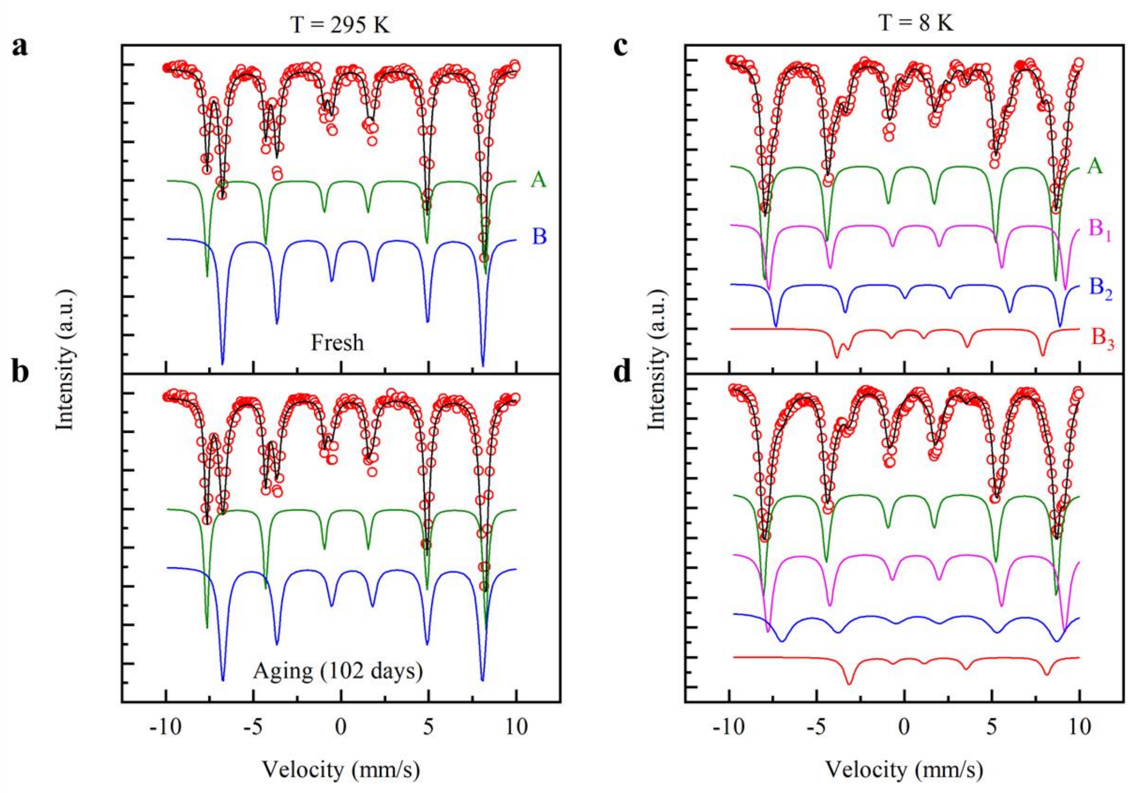

Supplementary Figure 7. The Mössbauer spectra were taken at 295 and 8 K for the fresh and oxidized (102 days) sample. (a,b) The spectra for the oxidized sample show similar peak positions to the fresh one, which indicates that the oxidized sample has mainly Fe_3O_4 characters. (c,d) The Mössbauer spectra were taken at 8 K for the fresh and oxidized (102 days) sample. The fitting for the spectra is done with a published method.[15,16] The similar structure of the spectra shows that the Verwey transition is preserved in the oxidized sample, and hence that it is still magnetite.

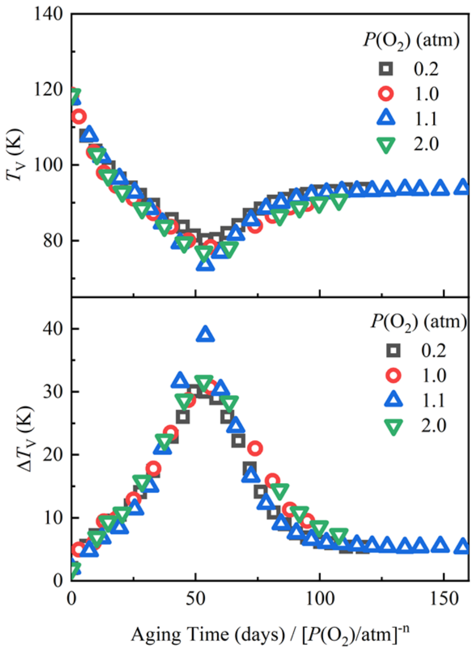

Supplementary Figure 8. The *P*(O_2) dependence of T_V and ΔT_V, defined as the FWHM of the peak, appeared in the *dM*/*dT*. The x-axis shows the scaled aging time defined as time/$P(O_2)^{-n}$, where n = 0.18 is the measured slope from the linear fitting as shown in the inset of Fig. 3b.

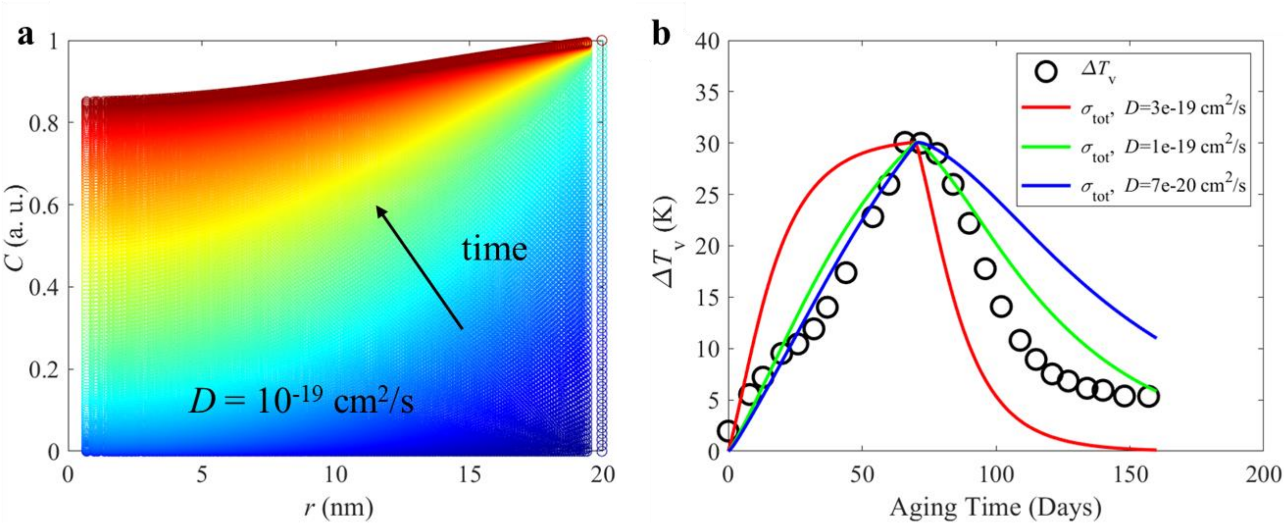

Supplementary Figure 9. Theoretical modeling results. (a) The simulated oxygen concentration profile C as a function of radius r from the diffusion equation (see Methods). (b) The calculated total strain σ_{tot} for three values of the diffusion coefficient.

Sample	Site	H_{hf} (kOe)	E_Q (mm/s)	IS (mm/s)
Fresh	A (Fe^{3+})	491.6 (0.00)	0.00 (0.01)	0.30 (0.01)
	B ($Fe^{2.5+}$)	459.8 (0.00)	0.02 (0.01)	0.66 (0.00)
Aging (102 days)	A (Fe^{3+})	492.8 (0.00)	-0.00 (0.01)	0.30 (0.00)
	B ($Fe^{2.5+}$)	458.6 (0.00)	0.03 (0.01)	0.65 (0.01)

Supplementary Table 1. The fitted parameters H_{hf}, E_Q, and IS from the Mössbauer spectra were taken at 295 K for the fresh and oxidized (102 days) sample. Estimated standard deviations are shown in parenthesis. The fitting method is the same as reported.[17]

**Sample**	**Site**	**H_{hf} (kOe)**	**E_Q (mm/s)**	**IS (mm/s)**
Fresh	A (Fe^{3+})	514.5 (0.00)	-0.07 (0.01)	0.36 (0.01)
	B1 (Fe^{3+})	523.9 (0.01)	0.06 (0.02)	0.69 (0.02)
	B2 (Fe^{2+})	502.4 (0.02)	-0.54 (0.04)	1.05 (0.02)
	B3 (Fe^{2+})	363.8 (0.02)	1.83 (0.04)	1.11 (0.02)
Aging (102 days)	A (Fe^{3+})	517.6 (0.01)	-0.07 (0.01)	0.35 (0.01)
	B1 (Fe^{3+})	524.2 (0.01)	0.04 (0.02)	0.67 (0.01)
	B2 (Fe^{2+})	485.9 (0.06)	0.10 (0.07)	0.81 (0.06)
	B3 (Fe^{2+})	351.2 (0.02)	2.21 (0.08)	1.36 (0.03)

Supplementary Table 2. The fitted parameters H_{hf}, E_Q, and IS from the Mössbauer spectra were taken at 8 K for the fresh and oxidized (102 days) sample. Estimated standard deviations are shown in parenthesis. The fitting method is reported elsewhere.[15,16]
